# Chronic exposure to MK-801 leads to olfactory deficits and reduced neurogenesis in the olfactory bulbs of adult male mice

**DOI:** 10.3389/fnbeh.2024.1441910

**Published:** 2024-09-05

**Authors:** Artem Sinegubov, Vyacheslav Dyachuk

**Affiliations:** Almazov National Medical Research Centre, Saint Petersburg, Russia

**Keywords:** neurogenesis, olfaction, MK-801, NMDA, schizophrenia

## Abstract

**Background:**

MK-801 is a drug widely used in preclinical studies to model schizophrenia in animals. Its distinctive feature is the ability to mimic pathological changes in social interactions. Unlike humans, rodents rely heavily on their sense of smell for social interaction. Since, as previously demonstrated, it also impairs neurogenesis, we set out to determine whether olfactory impairment is associated with chronic administration of the drug.

**Methods:**

The mice were divided into two groups, of which one was administered the drug for 3 weeks, and the other only once. Olfaction and social transfer of food preferences were tested after the drug administration period. At the end of the experiment, an immunofluorescence study was performed to determine differences in neurogenesis in the olfactory bulbs.

**Results:**

An olfactory deficit was observed in animals that received the drug for 3 weeks. These changes were also accompanied by an abnormal lack of food preference in the social transmission test. As a result of a morphological study, a pronounced decrease in the number of new neurons was found in the olfactory bulbs of the animals that had received the drug.

**Conclusion:**

Our results indicate that at least some of the impairments in social behavior of the animals exposed to NMDA receptor antagonists are likely caused by changes in the sense of smell. These changes are associated with disruptions of neurogenesis.

## 1 Introduction

NMDA receptor hypofunction is a well-known animal model of schizophrenia, especially effective in inducing negative symptoms. Several genetic and pharmacological approaches have been used to induce the NMDA receptor hypofunction in animal models of schizophrenia (Lee and Zhou, 2019). Exposure to MK-801 is widely used in rodent models due to such advantages as the simple study design and the relatively accurate induction of both negative and positive symptoms.

One of the crucial features of schizophrenia is the alteration of social interactions. MK-801 induces social withdrawal in rodents (Deiana et al., 2015; Mabunga et al., 2019) and has been proposed as a model for mimicking social impairment in schizophrenia and autism spectrum disorder. Furthermore, a significant amount of data that shows a important role of glutamatergic neurotransmission in the regulation of social behavior has been accumulated to date. Thus, knockout of the gene encoding NR1 in the forebrain leads to a decrease in motivation for social interaction and impairs social memory (Jacobs and Tsien, 2017). Mice heterozygous for a missense mutation in *Grin1* show abnormal anxiety-like and fear-related behaviors (Umemori et al., 2013). *Shank2−/−* mice with low NMDA receptor function exhibit autistic-like behavior with reduced social communication by ultrasonic vocalizations (Won et al., 2012). Some genetic variants of NMDA receptor are associated with emotional and social impairment in healthy adults (Lee et al., 2016), susceptibility for attention-deficit/ hyperactivity disorder (Dorval et al., 2007), and schizophrenia (Hall and Bray, 2022).

Mice rely heavily on olfaction during social interaction. The social transmission of food preference (STFP) is a well established, ethologically relevant test of olfactory memory in rodents. As was previously demonstrated, acute administration of MK-801 at high doses induces conditioned food aversions (Myhal and Fleming, 1990). The NMDA receptor was also shown to be important for regulating the olfactory nerve potentiation and the associated odor preference learning in neonatal rats (Schoppa et al., 1998). In adult mice, overexpression of *Grin2b* leads to improvement of olfactory memory (Schoppa et al., 1998).

On the other hand, NMDA receptor modulates postnatal neurogenesis in the subventricular zone (Lethbridge et al., 2012). The subventricular zone is a source of neuroblasts migrating and integrating into the olfactory bulbs. It was reported that functioning of the NMDA receptor plays a critical role in the survival of neuroblasts. Neuroblasts express functional NMDA receptors prior to entering the olfactory bulbs, and its tonic activation in an asynaptic environment promotes survival of neuroblasts during migration.

Taken together, we hypothesized that blockade of the NMDA receptor may impair neurogenesis in the olfactory bulbs and affect social interaction in mice.

## 2 Materials and methods

The care and use of animals were approved by the Ethics Committee of the Pavlov Institute of Physiology, Russian Academy of Sciences, and followed the guidelines of the EU Directive 2010/ 63/EU on the use of animals for scientific purposes.

### 2.1 Animals

Forty-eight male C57BL/6 J mice were obtained from the breeding colony of the Pavlov Institute of Physiology RAS. Animals aged 6 weeks at the starting point were used in the study. They were assigned to one of the two cohorts: (1) demostrators and (2) observers.

### 2.2 Treatments

(+)MK-801 (SigmaAldrich) and EdU (Lumiprobe) were dissolved in sterile saline at concentrations of 0.1 and 10 mg/mL, respectively. Fresh solutions were prepared on the day of injection and stored in a refrigerator between injections.

**Figure 1:**
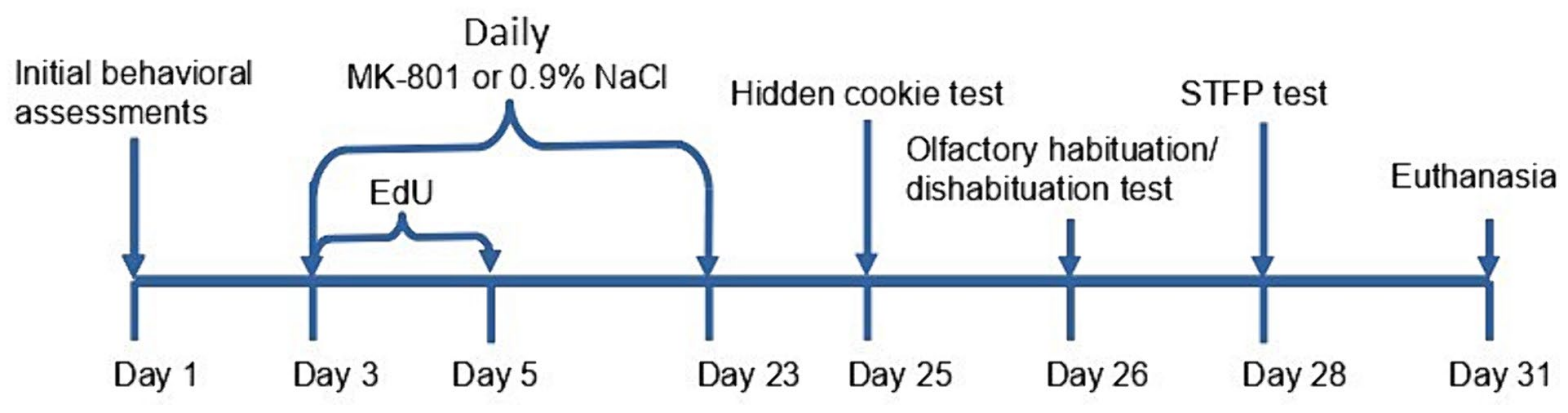
Experimental timeline for chronically treated animals. After the initial behavioral tests mice had received EdU for 3 days, and MK-801 or 0.9% saline for 3 weeks. Then a panel of tests were performed before the euthanasia after 28 days from the first injection of EdU.

A diagram of the treatment schedule is shown in Figure 1. Mice from the cohort of observers were randomly segregated into the following equal groups: (1) a vehicle control group comprising animals that received daily intraperitoneal injection of normal saline (0.9% NaCl) starting from Day 3 to Day 24; (2) a group chronically treated with MK-801, comprising animals that received daily intraperitoneal injections of 0.2 mg/kg MK-801 (injection volumes 40 to 60 μL) starting from Day 3 to Day 24; (3) a group acutely treated with MK-801, comprising animals that received daily intraperitoneal injection of normal saline (0.9% NaCl) starting from Day 3 to Day 23 and single intraperitoneal injection of MK-801 (0.2 mg/kg) on Day 24.

### 2.3 Behavioral tests

Cotton tip-based olfactory habituation/dishabituation test was based on the protocol of White and Youngentob (2004). Each mouse was allowed to acclimate for 30 min in the test cage. The test cage is a regular cage with an opaque glass tube inserted into the lid. The tube was used to present odor by inserting an applicator wetted with an odor solution. The use of the tube reduced the novelty-induced exploratory activity during changing the applicators. Different odors were presented to mice in the following order: water, cineol, and n-heptanol. Each odor was presented 3 times for 2 min. The inter-trial time was 1 min. Time of sniffing was measured for each run. Time of sniffing the novel odor and difference between the first and second presentations of the odor was calculated for further analysis.

For the hidden-cookie test, a piece of chocolate chip cookie was hidden in the same corner of the cage for all animals. Two days earlier, a piece of cookie was added to the food to familiarize the mice with the cookies. On the night prior to testing, the animals were deprived of food 4 h before the testing start time. A mouse was placed at the center of the cage and the time until it found the cookie was measured. If the cookie was not found within 10 min, the test was considered failed.

Social transmission of food preference was assessed according to previously published procedure (Wrenn, 2004) with minor modifications. In brief, the mice habituated to eat powdered chow were food-deprived for 18 h before the trial start. The demonstrators were randomly placed in individual cages with a jar of powdered food mixed with either 1% cinnamon or 2% cocoa for 1 h. After the jar was removed, the observer mouse was allowed to interact with the demonstrator for 30 min. During the interaction phase, the number of sniffs was recorded separately for sniffing the demonstrator’s muzzle and other parts of the body. After the interaction, the observers were caged individually for a 48-h period with two food jars: one jar contained the flavor of food eaten by the demonstrator (cued), and the other jar contained a novel flavor. Weight of the eaten food was recorded for each mouse.

The Fischer exact test was used to analyze the nominal data obtained from the hidden-cookie test. For all other datasets, a statistical analysis was performed using the one-way analysis of variance (ANOVA). Normality of data distribution was conformed using the Shapiro–Wilk’s test. *Post hoc* comparisons were performed using the Tukey’s range test and values were considered significant at *p* < 0.05.

### 2.4 Tissue preparation

On Day 31, the mice were killed by an overdose of isoflurane followed by transcardial perfusion of 4% paraformaldehyde (PFA) solution. Dissected brains were fixed in 4% PFA overnight, cryoprotected in 30% sucrose, embedded in optimal cutting temperature compound, and snap-frozen in a dry ice/acetone bath. Thin (30 μm) sections were cut through the entire olfactory bulb on a cryotome. Sections spaced 120 μm apart were selected, 4 sections per mouse. For antigen retrieval, free-floating sections were incubated in citrate buffer for 20 min at 96°C.

After permeabilization with 0.5% Tween-20 and blocking with 3% bovine serum albumin (BSA), sections were incubated with EdU staining solution (100 mM Tris, 1 mM CuSO_4_, 100 mM ascorbic acid, and 0.01 mM Alexa Fluor™ 488 Azide (Thermo Fisher Scientific, A10266)) for 30 min. After washing, the sections were incubated with primary antibodies overnight at 4°C, washed with phosphate buffered saline with Tween (PBST), and subsequently incubated with secondary antibodies and 4′,6-diamidino-2-phenylindole (DAPI) for 1 h at room temperature. The sections were mounted with a medium containing 90% glycerol and 10% PBS.

The following antibodies were used: Mouse Anti-NeuN (1,1,000, Abcam, ab104224), Rabbit Anti-Tyrosine Hydroxylase (1,1,000, Abcam, ab137869), Goat anti-Rabbit Secondary Antibody, Alexa Fluor™ 555 Goat anti-Mouse Secondary Antibody (Thermo Fisher Scientific, A-21428), and Alexa Fluor™ 647 (Thermo Fisher Scientific, A-21235).

### 2.5 Image acquisition and statistical analysis

Images were taken through a confocal microscope (LSM 710, Zeiss, Germany). Each section was scanned entirely with 10× to 40× objectives. The images were subjected to a blind analysis using ImageJ. EdU positive cell nuclei were identified and counted automatically with manual control using the ImageJ plugin ITCN (Schneider et al., 2012). Verification of predominant staining of neurons by counting double-positive cells was done manually. Statistical analysis was performed using one-way ANOVA. All data were tested for normality of data distribution using the Shapiro–Wilk’s test. *Post hoc* comparisons using the Tukey’s range test was performed and values were considered significant at *p* < 0.001.

## 3 Results

We tested olfactory sensitivity at two points, as indicated in Figure 1. The hidden-cookie and olfactory habituation/dishabituation tests were set up both before and after treatment. There was no difference between groups in both tests before treatment at Day 1 (data not shown).

**Figure 2:**
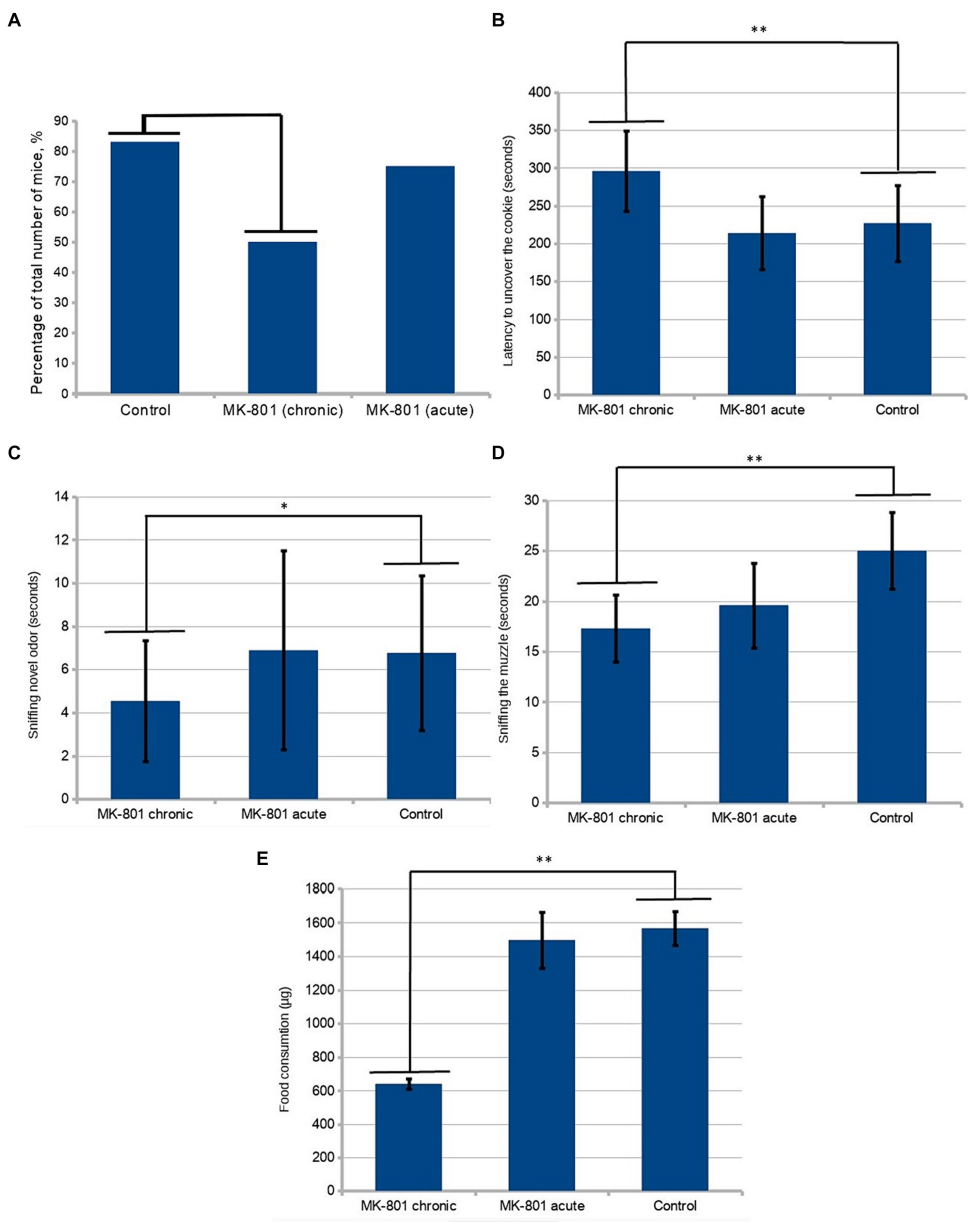
Behavioral assessment of olfaction. **(A)** A lower percentage of chronically treated animals than control mice were found in the hidden-cookie test (50 vs. 83%; *n* = 12, *p* = 0.193 in the Fisher exact test). **(B)** Mice exhibited longer latency to uncover the cookie after chronic treatment with MK-801. **(C)** In the olfactory habituation/dishabituation test, the chronically treated mice spent significantly less time sniffing novel odor. **(D)** Chronically treated mice tended to sniff the demonstrator’s muzzle less frequently. **(E)** After chronic exposure to MK-801 mice had significant reduction of preference to the preexposed food. * indicates *p* < 0.05 in Tukey *post hoc* test, ** indicates *p* < 0.01 in Tukey *post hoc* test. Bars represent mean ± SEM.

The drug causes a significant increase in locomotor activity. In order to avoid the influence of this effect on the results of behavioral tests, the next round of testing was set up 36 h after the last injection of the drug (Day 25). Locomotor activity was estimated as the total distance moved during the period of acclimation to the test cage. To test olfactory sensitivity, the hidden-cookie test was performed. Chronically treated mice were less likely to find the cookies (50 vs. 83%; *n* = 12, *p* = 0.193, Figure 2A). The latency to uncover the cookie was significantly longer in the MK-801-treated mice (227 ± 50.4 vs. 296 ± 53.2 s; *n* = 12, *p* < 0.05, Figure 2B). These changes were not observed in animals that received a single injection, 75% of mice found the hidden cookie with a mean latency of 214 ± 47.9 s.

In the olfactory habituation/dishabituation test, the chronically treated mice spent significantly less time sniffing novel odor (4.54 ± 2.79 vs. 6.77 ± 3.58, *n* = 24, *p* < 0.05) (Figure 2C). There was a slight tendency toward a decrease in the time of sniffing the control solution (water) in the animals that received the drug, which, however, was not statistically significant.

Afterwards, we tested the formation of food preference in mice. This test is ethologically relevant method to assess both olfactory memory and social behavior in these animals. The test exploits a naturally occurring food neophobia. It consists of three phases: (1) pre-exposure to novel food of the demonstrator; (2) interaction between the demonstrator and an observer; (3) testing the food preference of the observer. Normally, the observer mouse prefers the food eaten by its respective demonstrator.

Twelve animals from different litters were used as “demonstrators.” They were pre-exposed to cinnamon- or cocoa-flavored food. One demonstrator was allowed to interact with one observer from each group. Food preferences were determined by subsequent 48-h intakes of the novel food. During the interaction period, the demonstrators were placed in a wire cup-like container. The observers from both treated groups spent significantly less time in the area near the demonstrator. The near-demonstrator area was defined as an area with a radius of 5 cm around the wire cup-like container with the demonstrator in it. An analysis of frequency of sniffing using one-way ANOVA detected significant differences between the groups (*p* < 0.05, Figure 2D). Using the Tukey’s range test, it was found that chronically treated mice showed a significant reduction in the time of sniffing the demonstrator’s muzzle (24.83 ± 3.86 vs. 17 ± 3.36, *p* = 0.001076). We assessed the number of observer-demonstrator contacts, defined as the observer’s muzzle being in close proximity (less than 0.5 cm) to the demonstrator but without sniffing the demonstrator’s muzzle. A less pronounced, although statistically significant, difference between control and both groups receiving MK-801 was found in the number of contacts with the demonstrator mouse (19.3 ± 4.69 vs. 13.17 ± 5.22 and 19.3 ± 4.69 vs. 13.92 ± 4.78, *p* < 0.05).

A *post hoc* analysis to compare relative food consumptions during preference testing showed a reduced preference for the pre-exposed food in the animals chronically treated with the drug (1,567 ± 99.64 vs. 641 ± 31.92 μg, *p* < 0.01, Figure 2E). Also, these animals consumed less food in total. Although the mice that received a single injection exhibited reduced motivation to contact the demonstrators, they did not show an abnormal preference compared to control.

**Figure 3:**
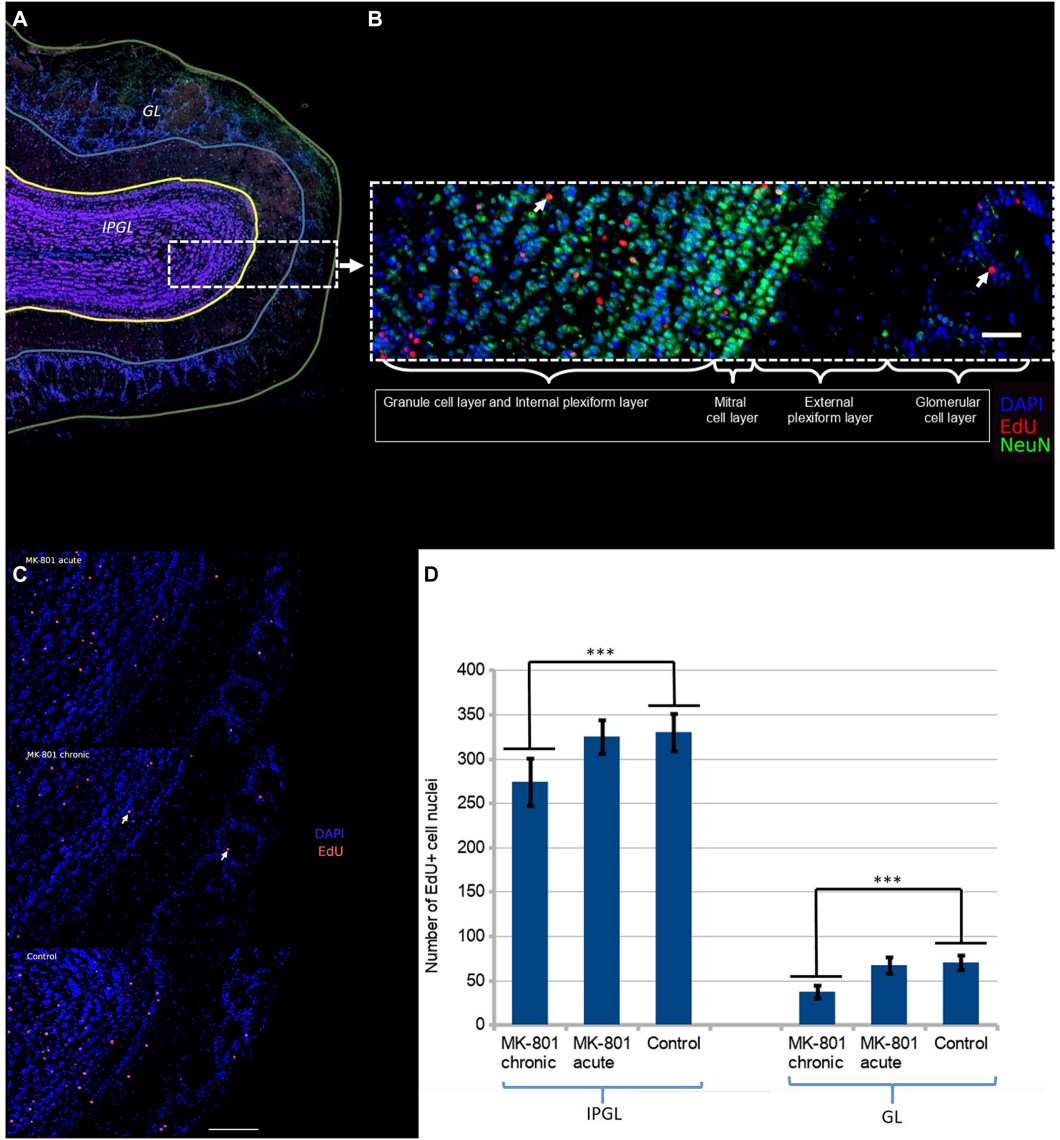
**(A)** Two regions of the olfactory bulb were selected for immunofluorescent assessment of neurogenesis: the glomerular layer (GL) and the granule and the internal plexiform layers (IPGL). **(B)** The newborn neurons predominantly populate the granular cell layer. The white arrow points the double-positive cell nucleus (NeuN+/EdU+). Scale bar is 100 μm. **(C)** Density of EdU-positive cell nuclei in olfactory bulb of each group. The white arrow points the double-positive cell nucleus (DAPI+/EdU+). Scale bar is 100 μm. **(D)** Significantly reduced number of EdU+ cells were observed in mice treated with MK-801 for 3 weeks. The left and right columns represent mean total number of EdU-positive cells per section in IPGL and GL, respectively. Sixty sections were analyzed in each group. *** indicates *p* < 0.001 in Tukey *post hoc* test. Bars represent mean ± SEM.

We further hypothesized that these changes may be associated with aberrant neurogenesis. We administered EdU to mice once a day for three consecutive days starting from Day 1 (50 mg/kg/day). The mouse brains were harvested on Day 31. The majority of newborn neurons differentiated into interneurons in the granule cell layer (Figure 3A). Initially, we hypothesized that behavioral changes might be due to aberrant neurogenesis in the glomerular layer. There are two populations of interneurons in the glomerular layer: dopaminergic and calretinin interneurons (Korshunov et al., 2020). Dopaminergic interneurons inhibit glutamate release from presynaptic terminals of the olfactory sensory neurons and thus, they reduce sensitivity and modulate the discrimination of olfactory signals. However we found that only a small number of neurons were positive to both tyrosine hydroxylase (TH+) and EdU. To ascertain whether there was a difference in the rate of integration of newborn neurons in the olfactory bulbs, we counted EdU positive cell nuclei in two regions: (1) the glomerular layer and (2) the internal plexiform and the granule cell layer (Figure 3B).

A significant reduction in the number of EdU+ neurons in the olfactory bulbs was found in the mice that had received MK-801 for 21 days (Figure 3C). The differences were more pronounced in the glomerular layer (70 ± 8.43 vs. 38 ± 6.67, *p* < 0.001, Figure 3D) than in the granule and internal plexiform layers (331 ± 21.39 vs. 275 ± 27.05, *p* < 0.001, Figure 3D). This effect was not observed in animals that had received a single injection both in the glomerular layer (70 ± 8.43 vs. 66,97 ± 8.75, Figure 3D) and in the granule and internal plexiform layers (331 ± 21.39 vs. 326 ± 19.39, Figure 3D).

## 4 Discussion

Based on multiple reports of abnormal social behavior of mice after administration of NMDA receptor antagonists, we hypothesized that these changes may be associated with altered olfaction. MK-801 is one of the drugs most widely used to induce psychosis-like phenotype in rodents. It has also been used in preclinical development of new antipsychotics. A substantial barrier to antiphsychotic drug development is the lack of suitable animal models that accurately mimic the disease. Hence, it is critically important to interpret accurately the behavioral changes in the model animals.

We have found that the changes in social behavior observed after the chronic administration of MK-801 are, at least in part, due to the impairment of the olfactory system. These may be associated with disruption of synaptic transmission in the olfactory system.

Social transmission of food preference is usually interpreted as a functional indicator of olfactory memory. Glutamatergic neurotransmission plays a significant role in memory. Indeed, it has been demonstrated that rats after a single injection of MK-801 show an increase in the amount of novel food consumed and this effect is reversible by the administration of the antipsychotic quetiapine (Mutlu et al., 2017). However, this effect was relatively small. We have observed that reduced avoidance of novel food is significantly more pronounced in mice repeatedly treated with MK-801. This phenomenon cannot be attributed to blockade of NMDA receptors since the test was performed 36 h after last administration of the drug.

This observation is consistent with previously published data suggesting that over-expression of NMDA-NR2B leads to increased food preference. This effect was interpreted as an improvement in memory (White and Youngentob, 2004). In keeping with this study, it was found that MK-801 impairs social memory by affecting glutamatergic neurotransmission in forebrain (Zhang et al., 2022).

On the other hand, knockout of NMDA receptors leads to a dramatic decrease in neuroblast survival. The majority of neuroblasts born in the postnatal subventricular zone migrate to the olfactory bulb where they integrate into existing neural circuits. Thus, we hypothesized that a suppression of neurogenesis due to NMDA receptor blockade may be an additional factor influencing behavior in the STFP test. As expected, we found that there is a significant olfactory deficit in mice after chronic exposure to MK-801. We previously found that MK-801 also disrupts the functioning of the peripheral part of the olfactory sensory system, the olfactory neuroepithelium (Bigdai and Sinegubov, 2022). The sense of smell plays a key role in the social behavior of rodents. Taken together, these findings suggest that some of the effects of chronic administration of NMDA receptor antagonists may be interpreted as sensory rather than behavioral disturbances.

It is controversial whether olfactory impairment is part of the endophenotype of schizophrenia. It has been shown that odor detection thresholds are significantly increased in patients with schizophrenia, and, consequently, the olfactory sensitivity is weakened (Rioux et al., 2005). There are also reports that the olfactory bulbs in people with schizophrenia exhibit a significant reduction in the expression of proteins associated with glutamatergic neurotransmission (Egbujo et al., 2015). Olfaction is closely associated with the frontal and temporal brain regions, which are among the most affected regions in schizophrenia. Further studies are needed to determine whether the phenomena observed in patients with this mental disorder and in the animal model overlap.

## Data Availability

The raw data supporting the conclusions of this article will be made available by the authors, without undue reservation.
